# miR-4454 Promotes Hepatic Carcinoma Progression by Targeting Vps4A and Rab27A

**DOI:** 10.1155/2021/9230435

**Published:** 2021-11-03

**Authors:** Haoming Lin, Rui Zhang, Wenrui Wu, Liming Lei

**Affiliations:** ^1^Department of Pancreato-Biliary Surgery, Sun Yat-Sen Memorial Hospital, Sun Yat-Sen University, No. 107 Yanjiang West Road, Guangzhou, 510120 Guangdong, China; ^2^Department of Intensive Care Unit of Cardiovascular Surgery, Guangdong Cardiovascular Institute, Guangdong Provincial People's Hospital, Guangdong Academy of Medical Sciences, Laboratory of South China Structural Heart Disease, Guangzhou, 510080 Guangdong, China

## Abstract

Hepatocellular carcinoma (HCC) has high morbidity and mortality. MicroRNAs (miRNAs), which could be regulated by cancer-derived exosomes, play critical regulatory roles in the initiation and development of cancer. However, the expressions, effects, and mechanisms of abundant miRNAs regulated by HCC cancer-derived exosomes in HCC remain largely unclear. Exosomes of HepG2 cells under heat shock, TGF-*β*1, doxorubicin, acid and hypoxia/reoxygenation (H/R) conditions, and exosomes were successfully identified by transmission electron microscopy and Western blot analysis. The identified exosomes were then applied to evaluate the miRNA expression profiles by RNA sequencing. Mechanically, we discovered that doxorubicin was upregulated, TGF-*β*1 downregulated the expressions of Vps4A, Rab27A, Alix, and Hrs in HepG2 cells and exosomes, and Vps4A and Rab27A, as target genes for miR-4454, could also be downregulated by miR-4454. Functionally, we revealed that miR-4454 inhibitor and miR-4454 inhibitor-mediated exosomes could markedly suppress proliferation, migration, invasion, and vascularization and accelerate cycle arrest, apoptosis, and ROS of HepG2 cells. This study provided many potential HCC cancer-derived exosome-mediated miRNAs in HCC under 5 different stimulus conditions. Meanwhile, we certified that miR-4454 in exosomes could provide a novel and effective mechanism for HCC function.

## 1. Introduction

Cancer is one of the most terrible diseases in modern society [1]. According to research estimates, there were 18.1 million new cancer cases and 9.6 million cancer deaths worldwide in 2018 [2, 3]. Among them, hepatocellular carcinoma (HCC) ranks seventh among all types of cancer [4]. In China, HCC is one of the most widespread malignancies and one of the leading causes of cancer death, accounting for more than 50% of new cases and deaths worldwide [5]. Currently, the therapy of HCC is still mainly surgery by removing liver lesions to improve the survival rate [6]. However, survival rates for patients with HCC remain low. Sorafenib, as a multikinase inhibitor, is the first approved drug for the treatment of advanced HCC, which cannot be surgically removed worldwide [7]. However, sorafenib has the disadvantage of a slow curative effect and large side effects. In addition, the drug in combination with chemotherapy and radiation therapy will cause serious adverse reactions, such as myelosuppression, hypoimmunity, and a decline in white blood cells [8]. Therefore, it is urgent to study and develop new drugs to treat HCC.

Exosomes are bilayer-coated vesicles released by cells under physiological and pathological conditions [9]. Exosomes have been discovered to carry a range of bioactive substances, including DNA, microRNA (miRNA), lncRNAs, mRNA, proteins, and lipids [10]. Exosomes have been reported to affect various biological processes, such as tumor immunity, tumor invasion and metastasis, chemotherapy resistance, and angiogenesis through different molecular mechanisms [11, 12]. Several studies have confirmed that exosomes can affect the progression of multiple cancers [13–15]. Exosomes have become a novel mechanism to regulate the phenotype of tumor behavior [10]. Therefore, a detailed exploration of the relevant mechanisms of exosomes in the malignant phenotype of HCC could provide new strategies for the diagnosis and intervention of HCC.

Furthermore, tumor cells have been proven to produce many exosomes, which have significant effects on both the tumor microenvironment and tumor immunity [16]. Tumor-derived exosomes are rich in miRNAs, which are often more abundant than derived cells. miRNAs are short noncoding RNAs with approximately 19-22 nucleotides, which can play vital regulatory roles in numerous biochemical activities of cells [17, 18]. With the advancement of research, evidences revealed that miRNAs are closely related to the development of HCC, such as miR-96-5p [19], miR-612 [20], miR-130b [21], miR-30e-3p [22], and miR-802 [23]. Therefore, analysis of the miRNA expression profile in exosomes of HCC cells has become a new direction for the study of the biological behavior of HCC, which could provide a novel idea for the early diagnosis and prognosis of HCC.

Recent research suggested that under stress conditions such as antitumor drugs, the phenotype of exosomes released by HCC cells could be changed, and the surface of exosomes is highly expressed with shock proteins [24, 25]. In addition, these exosomes can significantly enhance the killing activity of NK cells [24, 25]. Therefore, changes in the internal and external environment can cause changes in tumor-derived exosome-packed molecules. In the present study, we further explored the effects of different stimuli (heat shock, TGF-*β*1, doxorubicin, and acidic and hypoxia/reoxygenation (H/R)) on exosomes that regulate the miRNA expression profile. Meanwhile, we investigated whether exosome-mediated miRNAs are a novel self-regulating and protective mechanism of HCC. Furthermore, we explore the underlying mechanisms and functions of miR-4454 in HCC and the influences of miR-4454 inhibitor-mediated exosomes on proliferation, cycle, apoptosis, ROS, migration, invasion, and vascularization of HCC.

## 2. Materials and Methods

### 2.1. Cell Culture and Treatment

HepG2 and HEK293T cells were obtained from ATCC. Cells were cultured in Dulbecco's modified Eagle medium (DMEM) with 10% fetal bovine serum (FBS, Thermo Fisher Scientific, Inc. China) at 37°C in 5% CO_2_. When the cells were in the logarithmic growth phase, they were used for experiments. HepG2 cells were also treated with doxorubicin, H/R, acidic, TGF-*β*1, or heat shock, respectively.

### 2.2. Cell Transfection

The miR-4454 inhibitor and the negative control (NC) were obtained from GenePharma (Shanghai, China). HepG2 cells (1 × 10^5^ cells/well) were inoculated in 6-well plates and transfected with the miR-4454 inhibitor and NC using Lipofectamine 3000 reagent (Invitrogen) according to experimental instructions.

### 2.3. Exosome Extraction

According to a previous study, exosomes were extracted by centrifugation [26]. HepG2 cells in each group were kept in exosome-free medium with FBS. The culture medium was used to separate exosomes by ultracentrifugation at 300 × g for 20 min, 3000 × g for 20 min, and then, the supernatant was centrifuged (10000 × g for 30 min at 4°C). After centrifugation, the supernatant was filtered using a 0.22 *μ*m membrane and then centrifugated at 120,000 × g for 70 min. Finally, the resuspended pellet in PBS was used to obtain exosomes by ultracentrifugation at 120,000 × g for 70 min at 4°C. The extracted exosomes were resuspended with PBS and stored at -80°C.

### 2.4. Electron Microscopy

The purified exosomes were resuspended with PBS and fixed with 2% paraformaldehyde. The mixture was then dropped onto EM grids. After drying, the exosomes were stained with 1% uranyl acetate and the grids were measured with the HT7700 transmission electron microscope (Hitachi, Tokyo, Japan).

### 2.5. Microarray and Hierarchical Clustering Analysis

This array was performed using the Glue Grant Human Transcriptome Array (GG-HTA, manufactured by Affymetrix Inc.) [27]. First, total RNAs were synthesized into cDNAs, and then, the cDNAs were fragmented, labeled, and hybridized to the Affymetrix GG-H Array. The treated slides were then scanned with the Affymetrix GeneChip Scanner 3000 7G (Affymetrix, Santa Clara, CA). Based on the previous report [28], the R language package was applied for hierarchical cluster analysis.

### 2.6. qRT-PCR Assay

Total RNAs were collected using Trizol reagent according to the instructions. The cDNAs were then synthesized by reverse transcription using the All-in-One TM First-Strand cDNA Synthesis Kit (GeneCopoeia, Guangzhou, China). Relative gene expressions were confirmed using the QuantiFast SYBR Green PCR Kit (Qiagen). The relative quantitative analysis was then counted using the 2^-△△Ct^ method. All primers were synthesized by Shanghai Sangon Bioengineering Co. Ltd. (Shanghai, China), and the primer sequences were also displayed in Table 1.

### 2.7. Western Blot Assay

RIPA lysate, including a protease inhibitor, was applied to extract the total proteins from treated HepG2 cells or exosomes. After centrifugation at 4°C, the supernatant was taken. After the protein concentration was determined by the BCA method, the total protein (40 *μ*g) in each group was separated by 12% SDS-PAGE electrophoresis and transferred into PVDF membranes. Then the target protein in membrane was sealed with 5% skim milk, and addressed with primary antibodies at 4°C overnight and the corresponding secondary antibodies for 2 h. After the reaction of the ECL reagent (Bio-Rad) for 5 min, the results were obtained by developing imaging in a dark room. And the primary antibodies contained CD63 (1 : 5,000, ab134045, Abcam), CD9 (1 : 2000, ab92726, Abcam), TSG101 (1 : 5,000, ab125011, Abcam), Calnexin-CNX (1 : 20,000, ab92573, Abcam), cleaved caspase-3 (1 : 500, ab32042, Abcam), Cyclin D1 (1 : 10000, ab134175, Abcam), Alix (1 : 1000, ab275377, Abcam), Hrs (1 : 1000, ab155539, Abcam), Rab27A (1 : 3000, ab55667, Abcam), Vps4A (1 : 3000, ab229806, Abcam), BAG5 (1 : 2000, ab97660, Abcam), TRPV6 (1 : 400, 39563, SAB), and GAPDH (1 : 10000, ab8245, Abcam).

### 2.8. Detection of ROS

The level of ROS in each group was tested using DCFH-DA based on the instructions of the ROS assay kit (Solarbio, China, Cat. No. CA1410).

### 2.9. Luciferase Reporter Gene Assay

WT-pmirGLO-Vps4A, Mut-pmirGLO-Vps4A, WT-pmirGLO-Rab27A, and Mut-pmirGLO-Rab27A were purchased from Hualian Biotechnology Co. Ltd. (Wuhan, China). HEK293T cells were cotransfected with the corresponding plasmids and miR-4454 mimics using Lipofectamine 3000 reagent (Invitrogen). After 48 h, the harvested cells were examined using the Dual-Luciferase Reporter Assay System (Promega).

### 2.10. CCK-8 Assay

The treated HepG2 cells (2 x 10^3^ cells/well) were collected and inoculated into 96-well plates 2 × 10^3^ with 6 multiple holes in each group. After the cells were attached to the wall, the medium was replaced with a new medium that included a 10 *μ*L CCK-8 solution (Beyotime, Haimen, China). After incubation for 2 h, the optical density (OD) value was measured using a microplate (BIOTEK, Vermont, USA) at 450 nm.

### 2.11. Colony Formation Assay

The treated HepG2 cells (1 × 10^3^ cells/well) were inoculated in a 6-well plates. The culture medium was changed every 3 days. After 2 weeks, clumps of cells appeared, and the culture was terminated. After washing with PBS, cells were fixed with 4% paraformaldehyde and then stained with 0.1% crystal violet dye (Sigma-Aldrich) for 15 min. After washing, the cells were photographed and counted.

### 2.12. EdU Staining

The treated HepG2 cells were added with 100 *μ*L complete medium including 50 *μ*mol/L EdU reagent in the EdU kit (Solarbio, Cat. No. CA1170) for 2 h. After washing, cells were fixed and decolored with 50 *μ*L glycine (2 mg/mL) for 5 min. The results were observed and photographed under the fluorescence microscope.

### 2.13. Flow Cytometer

Briefly, after culturing, treated HepG2 cells were harvested and washed twice with phosphate buffered saline (PBS). Next, the cells were fixed in 70% ethanol for 2 h and then incubated with annexin V-isothiocyanate (FITC) and propidium iodide (PI) (Keygentec, Nanjing, Jiangsu, China) for 10 min in the dark. Finally, flow cytometry (BD Biosciences, Franklin Lakes, NJ, USA) was used to analyze apoptosis. For cell cycle, cell cycle analysis kit (with RNase, Bioss, Cat. No. BA00205) was applied according to experimental instructions. After incubation for 15 min in darkness, apoptosis and cell cycle were examined by flow cytometry and the data were calculated with the CellQuest™ software (648089, Becton-Dickinson, USA).

### 2.14. Transwell Assay

For the migration assay, treated HepG2 cells (1 × 10^5^ cells/well) in serum-free medium were added into the superstratum of the Transwell chamber. 600 *μ*L medium with 10% FBS was added into the substratum of the Transwell chamber. After incubation for 24 h, cells were fixed with 70% methanol and stained with 4% crystal violet. The migrated cells were observed under a microscope, and cell counts were recorded. For the invasion assay, the diluted Matrigel (40 *μ*L) was prepaved in the Transwell chamber for 24 h.

### 2.15. Wound Healing Assay

The treated HepG2 cells were seeded in a 6-well plate. When the cell density reached 100%, a straight line was drawn vertically and evenly at the bottom using the sterilized yellow tip. After washing, they were replaced with a new serum-free medium and photographed. Scratch healing was observed under the microscope.

### 2.16. Statistical Analysis

All data from the experiment were displayed as mean ± SD. Data were calculated by using the SPSS software (ver. 20.0, SPSS, Inc., Chicago, USA). Statistical results were presented using GraphPad Prism 8.0. *p* < 0.05 was considered statistically significant.

## 3. Results

### 3.1. Identification of the Morphological Structure of Exosomes in Heat Shock, TGF-*β*1, Doxorubicin, Acidic, and H/R Conditions

**Figure 1:**
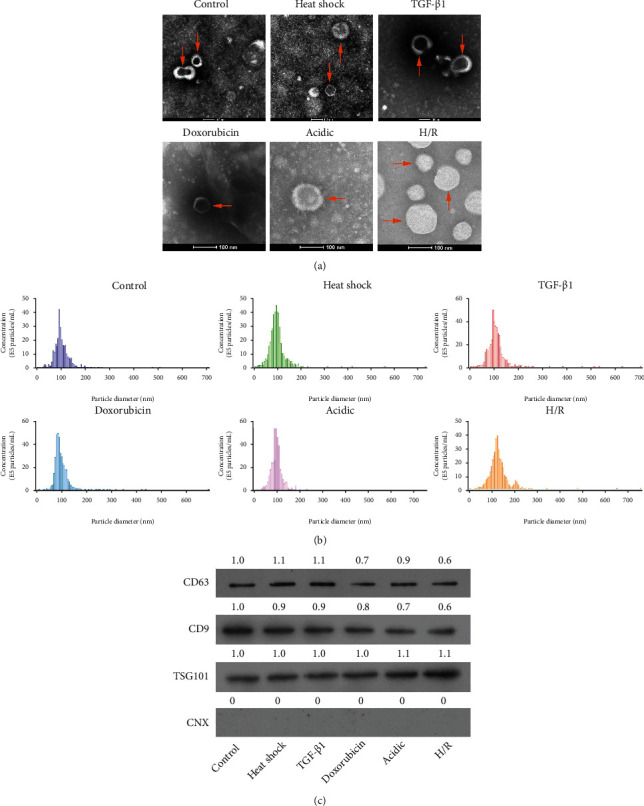
Morphological structure identification of exosomes in heat shock, TGF-*β*1, doxorubicin, acidic, and H/R conditions. (a) After heat shock, TGF-*β*1, doxorubicin, acidic, and H/R, the morphology of exosomes from HepG2 cell culture supernatants was assessed using TEM. Typical exosomal morphology is indicated by arrows. (b) The particle size distributions of exosomes were displayed. (c) Western blot was adopted to test exosome markers (CD63, CD9, and TSG101) and the endoplasmic reticulum indicator (CNX) in each group.

To investigate the properties of supernatant exosomes from hepatoma cells under the stimuli of different factors, HepG2 cells were stimulated with heat shock, TGF-*β*1, doxorubicin, acidic, and H/R, respectively. First, the supernatant exosomes were extracted from each group of HepG2 cells using the density gradient centrifugation method. TEM was applied to observe the morphology of exosomes in each group. The results showed that in the control group, the exosomes were uniform in size and round or elliptical with a complete envelope and typical double layer membrane structure; in the heat shock group, the exosomes had thin membranes, varied in size but had a clear background; in the TGF-*β*1 group, the exosomes were completely round or elliptical, containing internally uniform low electron density materials; in the doxorubicin groups, the exosomes had thin membranes, small sizes, and internally high electron density materials; in acidic groups, the exosomes had internally high electron density materials and many cell debris; in the H/R group, the exosomes had larger sizes and internally high electron density materials. And the overall particle sizes of 30-120 nm in each group were consistent with exosomes (Figure 1(a)). Besides, the particle size distributions of exosomes were further counted in each group. The results showed that the particle sizes of exosomes were about 100 nm in diameter, which conformed to the requirements of exosome particle size (Figure 1(b)). Furthermore, Western blot data certified that exosome markers (CD63, CD9, and TSG101) could be significantly expressed, while endoplasmic reticulum indicator (CNX, Calnexin) cannot be expressed in HepG2 cells of each group (Figure 1(c)). Therefore, our results revealed that the exosomes had been successfully extracted, and the morphology of the exosomes could be changed by different stimulating factors, such as heat shock, TGF-*β*1, doxorubicin, acidic, and H/R.

### 3.2. Length Distributions of miRNAs in Exosomes under Heat Shock, TGF-*β*1, Doxorubicin, Acidic, and H/R Conditions

**Figure 2:**
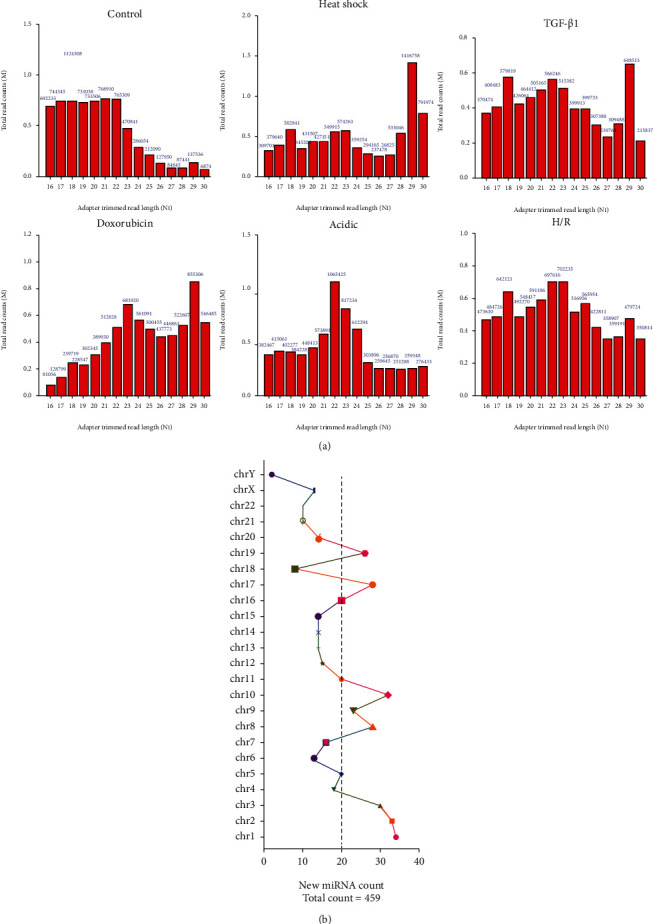
Length distributions of miRNAs in exosomes under heat shock, TGF-*β*1, doxorubicin, acidic and H/R conditions. HepG2 cells were stimulated under different conditions, including heat shock, TGF-*β*1, doxorubicin, acidic, and H/R, and miRNA expression profiles were evaluated by RNA sequencing in exosomes of HepG2 cell culture supernatants. (a) The length distributions of the miRNAs in each group were exhibited. (b) The distribution of miRNAs on each chromosome was shown.

RNA sequencing was carried out to further compare the expression differences of miRNAs in exosomes of HepG2 cells in different stimuli environments. In addition, the length distributions of miRNAs in each group were analyzed, and the results revealed that the length distributions of miRNAs were inconsistent in the 6 groups (Figure 2(a)). Meanwhile, we showed that newly discovered miRNAs with new differences (*n* = 459) were distributed primarily on chromosomes 1, 2, 3, 8, 9, 10, 17, and 20 (Figure 2(b)).

### 3.3. GO Enrichment Analyses of Upregulated miRNAs in Exosomes under Doxorubicin, H/R, Acidic, TGF-*β*1, and Heat Shock Conditions

**Figure 3:**
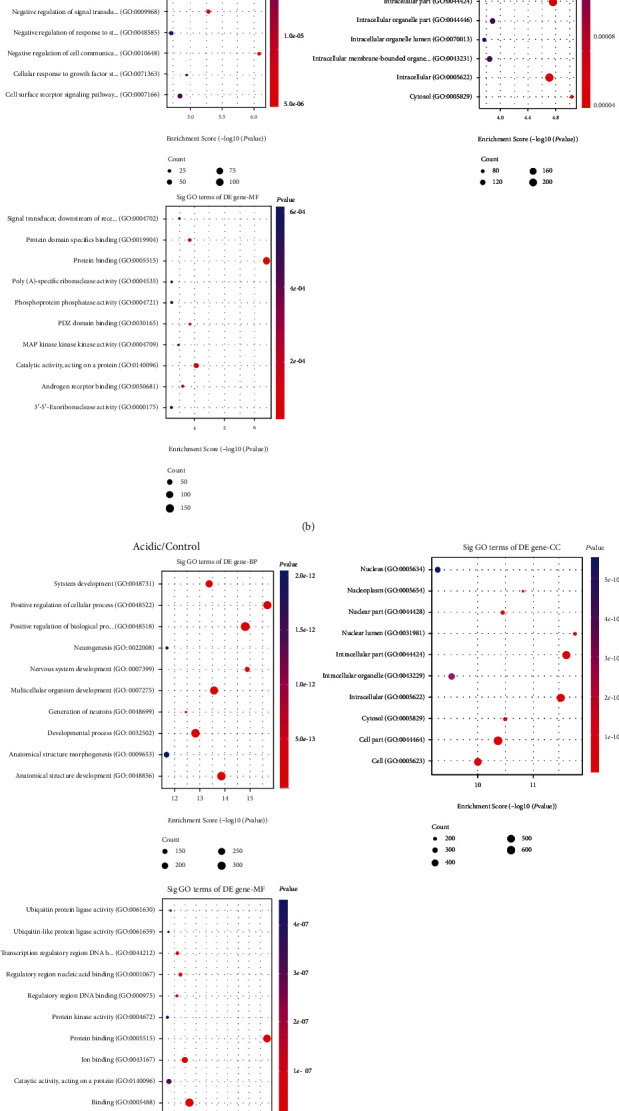
GO enrichment analyses of upregulated miRNAs in exosomes under conditions of doxorubicin, H/R, acid, and TGF-*β*1 conditions. GO enrichment of upregulated miRNAs was analyzed in exosomes from HepG2 cell culture supernatants after external stimuli, including (a) doxorubicin, (b) H/R, (c) acidic, and (d) TGF-*β*1.

**Figure 4:**
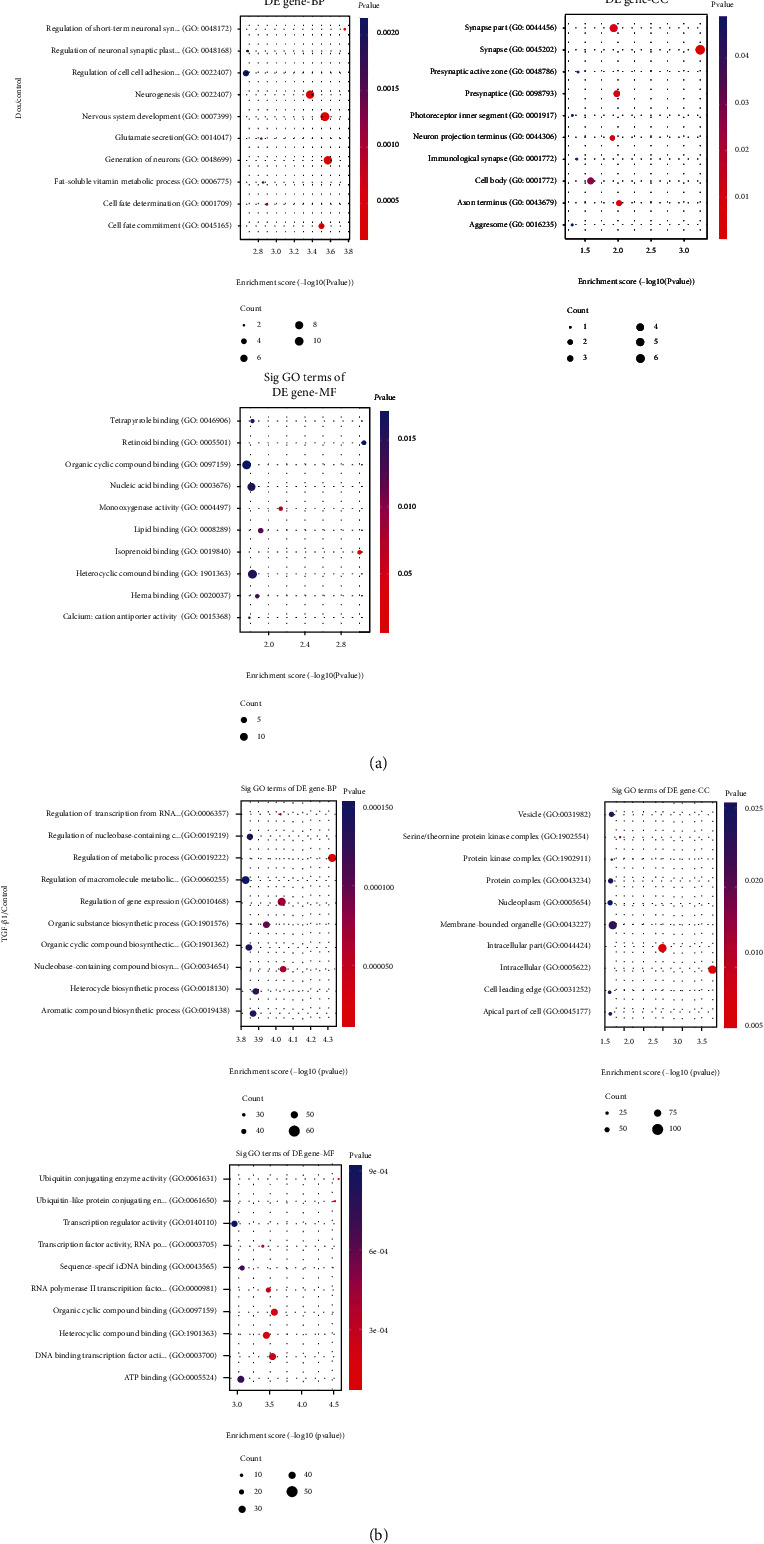
GO enrichment analyses of downregulated miRNAs in exosomes under doxorubicin and TGF-*β*1 conditions. GO enrichment of downregulated miRNAs was analyzed in exosomes from HepG2 cell culture supernatants after external stimuli, including (a) doxorubicin and (b) TGF-*β*1.

Based on the expression profiles of the upregulated miRNAs between control group and doxorubicin, H/R, acidic, or TGF-*β*1, GO analysis was performed. As the results showed, the upregulated miRNAs in the doxorubicin group were enriched in with respect to biological process (BP) terms (primary metabolic process (GO:0044238), organic substance metabolic process (GO:0071704), and nitrogen compound metabolic process (GO:0006807)), cellular component (CC) terms (intracellular part (GO:0044424), intracellular (GO:0005622), and cytoplasm (GO:0005737)), and molecular function (MF) terms (catalytic activity, acting on a protein (GO:0140096), RNA polymerase II regulatory region (GO:0000977), and RNA polymerase II regulatory region D... (GO:0001012)) (Figure 3(a)); the upregulated miRNAs in the H/R group were enriched in BP terms (regulation of metabolic process (GO:0019222), negative regulation of signaling (GO:0023057), and negative regulation of cell communication (GO:0010648)), CC terms (intracellular part (GO:0044424), intracellular (GO:0005622), and nucleus (GO:0005634)), and MF terms (protein domain specific binding protein (GO:0019904), protein binding (GO:0005515), and catalytic activity, acting on a protein (GO:0140096)) (Figure 3(b)); the upregulated miRNAs in acidic group were enriched in BP terms (developmental process (GO:0032502), positive regulation of biological processes (GO:0048518), and positive regulation of cellular process (GO:0048522)), CC terms (intracellular part (GO:0044424), intracellular (GO:0005622), and cell part (GO:0044464)), and MF terms (protein binding (GO:0005515), ion binding (GO:0043167), and binding (GO:0005488)) (Figure 3(c)); the upregulated miRNAs in TGF-*β*1 group were enriched in BP terms (system development (GO:0048731), developmental process (GO:0032502), and anatomical structure development (GO:0048856)), CC terms (plasma membrane part (GO:0044459), cytoplasm (GO:0005737), and intrinsic component of plasma membrane (GO:0031226)), and MF terms (core promoter proximal region sequence (GO:0000987), core promoter proximal region DNA bin... (GO:0001159), and calcium ion binding (GO:0005509)) (Figure 3(d)); the downregulated miRNAs in the doxorubicin group were enriched in BP terms (neurogenesis (GO:0022008), nervous system (GO:0007399), and generation of neurons (GO:0048699)), CC terms (synapse part (GO:0044456), synapse (GO:0045202), and presynapse (GO:0098793)), and MF terms (heterocyclic compound binding (GO:1901363), nucleic acid binding (GO:003676), and lipid binding (GO:0008289)) (Figure 4(a)); the downregulated miRNAs in TGF-*β*1 group were enriched in BP terms (regulation of metabolic process (GO:0019222), organic substance biosynthetic process (GO:1901576), and regulation of gene expression (GO:0010468)), CC terms (membrane-bounded organelle (GO:0043227), intracellular part (GO:0044424), and intracellular (GO:0005622)), and MF terms (organic cyclic compound binding (GO:0097159), heterocyclic compound binding (GO:1901363), and DNA binding transcription factor activity (GO:0003700)) (Figure 4(b)).

### 3.4. Validation of miRNAs with the Highest Differential Expressions

**Figure 5:**
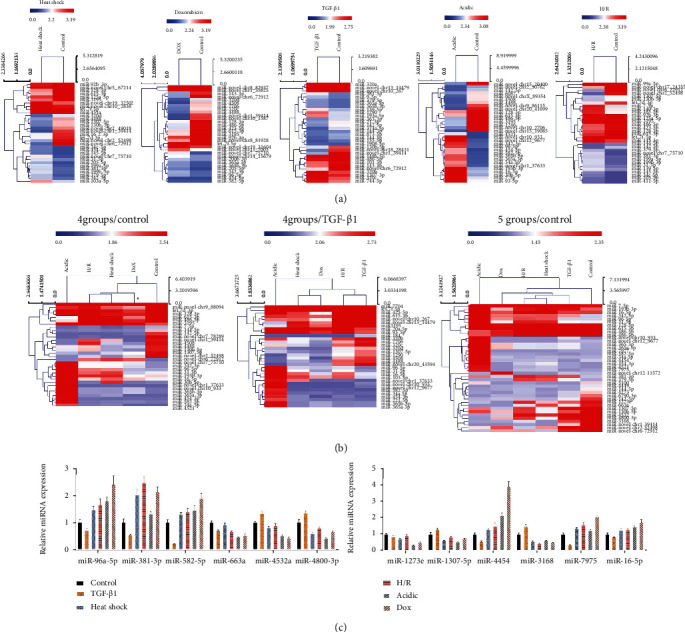
Validation of miRNAs with the highest differential expressions. After HepG2 cells were treated with heat shock, TGF-*β*1, doxorubicin, acidic, and H/R, exosomes were extracted from HepG2 cell culture supernatants. (a) miRNA expression distributions were displayed using heatmap between the heat shock, TGF-*β*1, doxorubicin, acidic, or H/R treatment groups and control group. (b) The heatmap showed the expressions of miRNAs among the heat shock, TGF-*β*1, doxorubicin, acidic, or H/R treatment groups and control group. (c) The top six upregulated miRNAs and the top six downregulated miRNAs were verified by qRT-PCR assay in each exosome group.

Next, top 16 upregulated miRNAs and top 16 downregulated miRNAs were screened according to the results of the RNA sequencing. We then adopted heatmap to show the expression distributions of miRNAs in the heat shock, TGF-*β*1, doxorubicin, acidic, or H/R treatment groups compared to the control group, respectively. As shown in Figure 5(a), red represents a high expression of miRNAs, and blue represents a low expression of miRNAs; differentially expressed miRNAs are listed on the right. Furthermore, we synthetically analyzed differentially expressed miRNAs in all groups. The miRNA expression profile was also shown using heatmap among heat shock, TGF-*β*1, doxorubicin, acidic, or H/R treatment groups and the control group (Figure 5(b)). Additionally, the top 6 upregulated and the top 6 downregulated miRNAs were selected and verified using the qRT-PCR assay in each exosome group. As shown in Figure 5(c), miR-96a-5p, miR-381-3p, miR-582-5p, miR-4454, miR-7975, and miR-16-5p were found to be significantly downregulated in the TGF-*β*1 group, signally upregulated in the heat shock, doxorubicin, acidic, and H/R groups relative to the control group; miR-4532, miR-4800-3p, miR-1307-5p, and miR-3168 were memorably upregulated in the TGF-*β*1 group, markedly downregulated in heat shock, doxorubicin, acidic, and H/R groups relative to the control group (Figure 5(c)). Based on the identification results, we discovered that miR-4454 expression was the most significant.

### 3.5. Vps4A and Rab27A, as Target Genes for miR-4454

**Figure 6:**
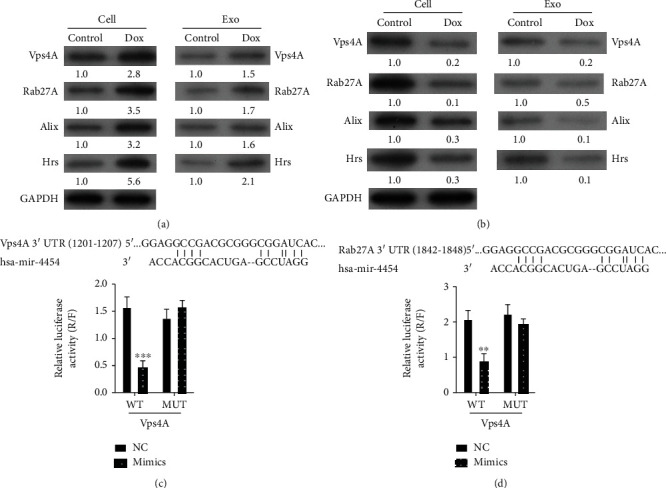
Vps4A and Rab27A, as miR-4454 target genes, were regulated by doxorubicin and TGF-*β*1 in HepG2 cells and exosomes. (a) After doxorubicin treatment, a Western blot analysis of Vps4A, Rab27A, Alix, and Hr was performed in HepG2 cells and exosomes. (b) After TGF-*β*1 treatment, Vps4A, Rab27A, Alix, and Hr levels were verified by Western blot assay in HepG2 cells and exosomes. (c, d) Binding sites between miR-4454 and Vps4A or Rab27A were predicted, and the regulations of miR-4454 in Vps4A and Rab27A were confirmed by the luciferase reporter gene assay.

Subsequently, we further explored the possible mechanism of miR-4454 in HepG2 cells and exosomes. HepG2 cells were stimulated with doxorubicin or TGF-*β*1, and exosomes were extracted. Our results of the Western blot analysis revealed that the expressions of Vps4A, Rab27A, Alix, and Hrs increased prominently in HepG2 cells and exosomes after treatment with doxorubicin versus the control group (Figure 6(a)). Meanwhile, we revealed that Vps4A, Rab27A, Alix, and Hrs expressions were markedly reduced in HepG2 cells and exosomes after treatment with TGF-*β*1 with respect to the control group (Figure 6(b)). Additionally, we also predicted possible binding sites between miR-4454 and Vps4A or Rab27A. To identify Vps4A and Rab27A that could bind to miR-4454, we constructed the vectors Vps4A and Rab27A with luciferase reporters, respectively (Figures 6(c) and 6(d)). Meanwhile, HepG2 cells were cotransfected with WT-Vps4A/Mut-Vps4A, WT-Rab27A/Mut-Rab27A, and NC/miR-4454 mimics. The results indicated that miR-4454 could significantly reduce the luciferase activities that could identify the interactions of miR-4454 and Vps4A or Rab27A in HCC (*p* < 0.01 and *p* < 0.001, Figures 6(c) and 6(d)).

### 3.6. Inhibition of miR-4454 Markedly Suppressed Proliferation and Accelerated Cycle Arrest, Apoptosis, and Oxidative Stress of HepG2 Cells

**Figure 7:**
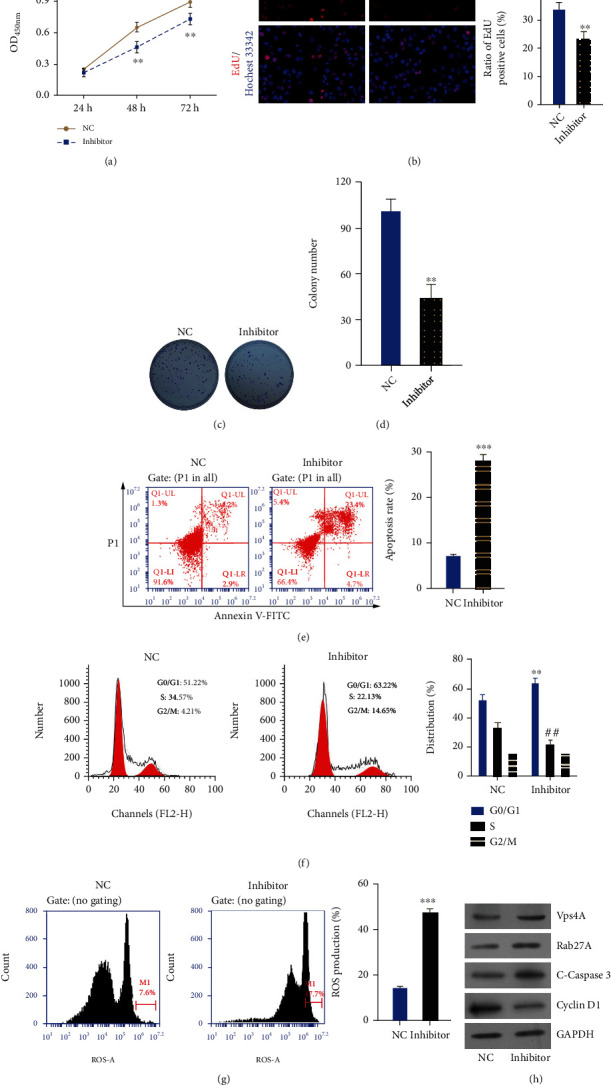
Inhibition of miR-4454 markedly suppressed proliferation and accelerated cycle arrest, apoptosis, and oxidative stress of HepG2 cells. HepG2 cells were transfected with the NC and miR-4454 inhibitor, respectively. (a–d) Cell proliferation was confirmed by CCK-8, EdU, and colony formation assays. (e) The apoptosis rate was determined by flow cytometry. (f) The cell cycle was examined by a flow cytometer. (g) ROS level was confirmed by the ROS assay kit. (h) Expressions of Vps4A, Rab27A, cleaved caspase-3, and Cyclin D1 were monitored by Western blotting. ^∗∗^*p* < 0.01 and ^∗∗∗^*p* < 0.001 vs. NC group; ^##^*p* < 0.01 for phase S versus NC group.

Next, we further demonstrated the influence of miR-4454 on the biological functions of HepG2 cells. First, our results from the CCK-8 assay showed that cell proliferation was dramatically reduced in the miR-4454 inhibitor group compared to the NC group (*p* < 0.01, Figures 7(a) and 7(b)). Similarly, the EdU and colony formation assay data revealed that miR-4454 inhibition had a prominent inhibitory effect on HepG2 cell proliferation (*p* < 0.01, Figures 7(b)–7(d)). Second, the experimental data from the flow cytometer showed that HepG2 cell apoptosis was markedly elevated in the miR-4454 inhibitor group compared to the NC group (*p* < 0.001, Figure 7(e)). Furthermore, our results determined that the number of HepG2 cells in phase G0/G1 increased observably, and the number of HepG2 cells in phase S decreased observably in the miR-4454 inhibitor group versus the NC group, indicating enhancement of cell cycle arrest in miR-4454 inhibitor-transfected HepG2 cells (*p* < 0.01, Figure 7(f)). Furthermore, our data indicated that ROS levels in HepG2 cells also increased prominently in the miR-4454 inhibitor group versus that of the NC group (*p* < 0.01, Figure 7(g)). And the results of the Western blot also indicated that Vps4A, Rab27A, and cleaved caspase-3 were upregulated, and Cyclin D1 was downregulated in the miR-4454 inhibitor group compared to that of the NC group (*p* < 0.001, Figure 7(h)). Thus, our results testified that inhibition of miR-4454 could dramatically prevent the malignant behavior of HCC.

### 3.7. Inhibition of miR-4454 Dramatically Inhibited the Migration, Invasion, and Vascularization of HepG2 Cells

**Figure 8:**
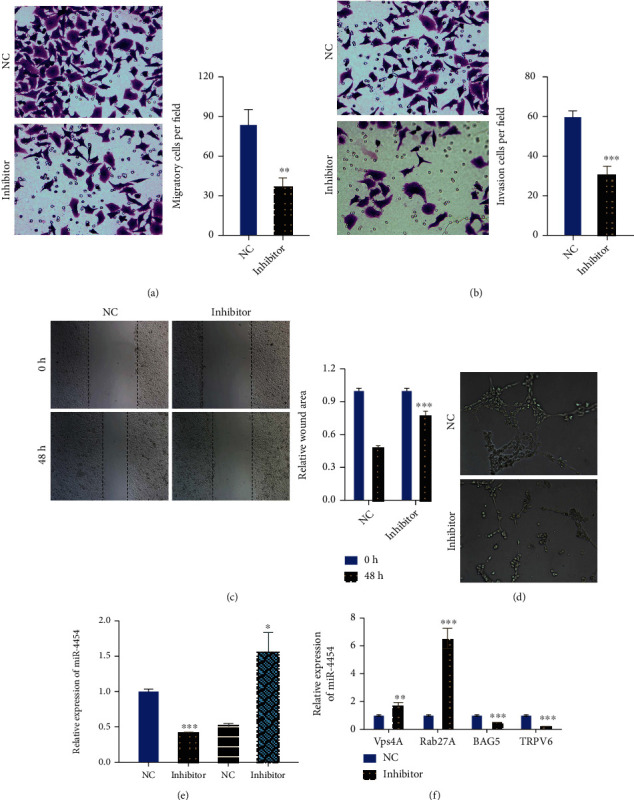
Inhibition of miR-4454 dramatically inhibited migration, invasion, and vascularization of HepG2 cells. NC and miR-4454 inhibitors were applied to transfect HepG2 cells. (a, b) The Transwell assay was used to evaluate cell migration and invasion. (c) A wound healing assay was adopted to identify the ability of cells to migrate. (d) The angiogenic ability of cells was evaluated on Matrigel. ^∗∗^*p* < 0.01 and ^∗∗∗^*p* < 0.001. (e) The expression of miR-4454 was examined by a qRT-PCR assay in HepG2 cells and exosomes after transfection with the miR-4454 inhibitor. ^∗^*p* < 0.05 and ^∗∗∗^*p* < 0.001. (f) The levels of the downstream genes (Vps4A, Rab27A, TRPV6, and BAG5) were monitored by applying the qRT-PCR assay in HepG2 cells transfected with the NC and miR-4454 inhibitor, respectively. ^∗∗^*p* < 0.01 and ^∗∗∗^*p* < 0.001.

Meanwhile, our results from the Transwell assay testified that inhibition of miR-4454 prominently weakened the migration and invasion capacities of HepG2 cells (*p* < 0.01 and *p* < 0.001, Figures 8(a) and 8(b)). Simultaneously, the results of the wound healing assay further verified that inhibition of miR-4454 had a strong suppressive role in HepG2 cell migration (*p* < 0.001, Figure 8(c)). Additionally, our results revealed that inhibition of miR-4454 signally inhibits the angiogenic ability of HepG2 cells (Figure 8(d)). Taken together, our results further confirmed the inhibitory function of the miR-4454 inhibitor on the malignant behavior of HCC. Furthermore, HepG2 cells were transfected with NC and miR-4454 inhibitor, and miR-4454 expression was certified in HepG2 cells and exosomes. We found that miR-4454 inhibitor significantly decreased miR-4454 expression in miR-4454 inhibitor-transfected HepG2 cells and notably increased miR-4454 expression in exosomes, suggesting the successful transfection of miR-4454 inhibitor in HepG2 cells (*p* < 0.05 and *p* < 0.001, Figure 8(e)). Our results also discovered that inhibition of miR-4454 markedly elevated the expressions of Vps4A and Rab27A and markedly reduced the expressions of TRPV6 and BAG5 in HepG2 cells (*p* < 0.01 and *p* < 0.001, Figure 8(f)).

### 3.8. miR-4454 Inhibitor-Mediated Exosomes Prominently Prevented Proliferation and Facilitated Cycle Arrest, Apoptosis, and Oxidative Stress in HepG2 Cells

**Figure 9:**
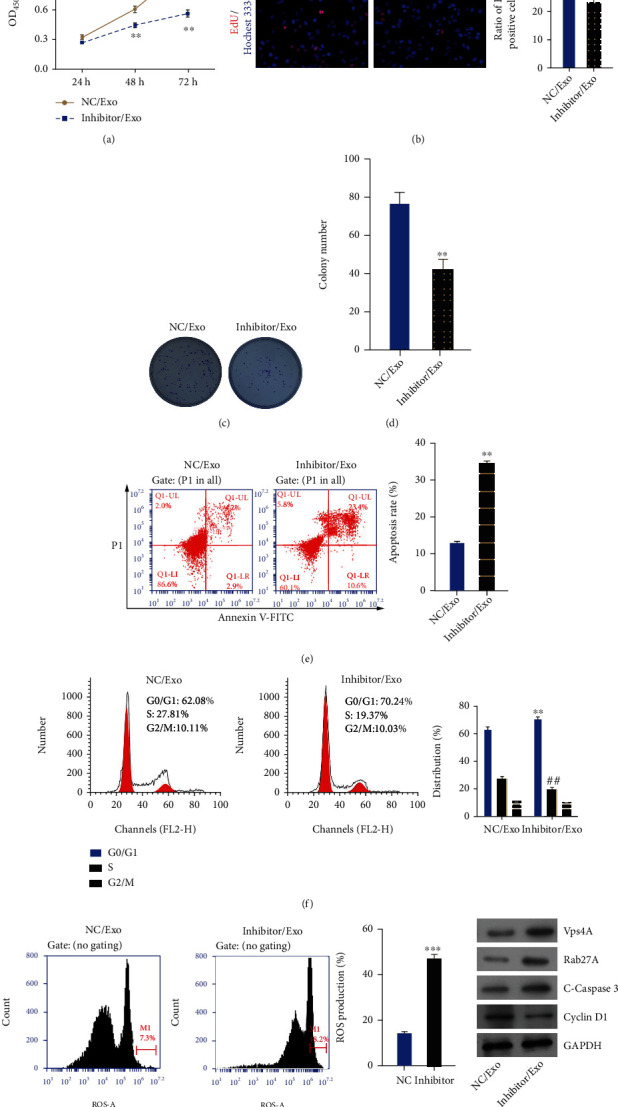
miR-4454 inhibitor-mediated exosomes prominently prevented proliferation and facilitated cycle arrest, apoptosis, and oxidative stress of HepG2 cells. Exosomes were extracted from NC or miR-4454 inhibitor-transfected HepG2 cells, and then, exosomes were applied to treat HepG2 cells. (a–d) CCK-8, EdU, and colony formation assays were adopted to examine the impacts of miR-4454 inhibitor-mediated exosomes on HepG2 cell proliferation (e, f). Cell apoptosis and cell cycle were surveyed by flow cytometry. (g) The ROS assay kit was used to test ROS level. (h) Western blot was utilized to determine the expressions of Vps4A, Rab27A, cleaved caspase-3, and Cyclin D1. ^∗^*p* < 0.05, ^∗∗^*p* < 0.01, and ^∗∗∗^*p* < 0.001 vs. NC/Exo group; ^##^*p* < 0.01 for phase S vs. NC/Exo group.

Subsequently, we further investigated miR-4454 inhibitor-mediated exosomes on the biological processes of HepG2 cells. We extracted exosomes from NC or miR-4454 inhibitor-transfected HepG2 cells, and HepG2 cells were treated with the extracted exosomes, respectively. Similarly, our experimental results of the CCK-8, EdU, and colony formation assays demonstrated that miR-4454 inhibitor-mediated exosomes could inhibit HepG2 cell proliferation (*p* < 0.05 and *p* < 0.01, Figures 9(a)–9(d)). Simultaneously, we discovered that apoptosis of HepG2 cells was markedly enhanced in miR-4454 inhibitor-mediated exosomes compared to NC-mediated exosomes (*p* < 0.01, Figure 9(e)). Furthermore, we revealed that miR-4454 inhibitor-mediated exosomes could markedly accelerate HepG2 cell cycle arrest (*p* < 0.01, Figure 9(f)). In addition, we discovered that miR-4454 inhibitor-mediated exosomes could also signally elevate ROS levels in HepG2 cells (*p* < 0.001, Figure 9(g)). And the results of Western blot showed that miR-4454 inhibitor-mediated exosomes could also result in upregulation of Vps4A, Rab27A, and cleaved caspase-3 and downregulation of Cyclin D1 in HepG2 cells (Figure 9(h)). In summary, we testified that miR-4454 inhibitor-mediated exosomes could also prevent the malignant activities of HepG2 cells.

### 3.9. miR-4454 Inhibitor-Mediated Exosomes Significantly Prevented Migration, Invasion, and Vascularization of HepG2 Cells

**Figure 10:**
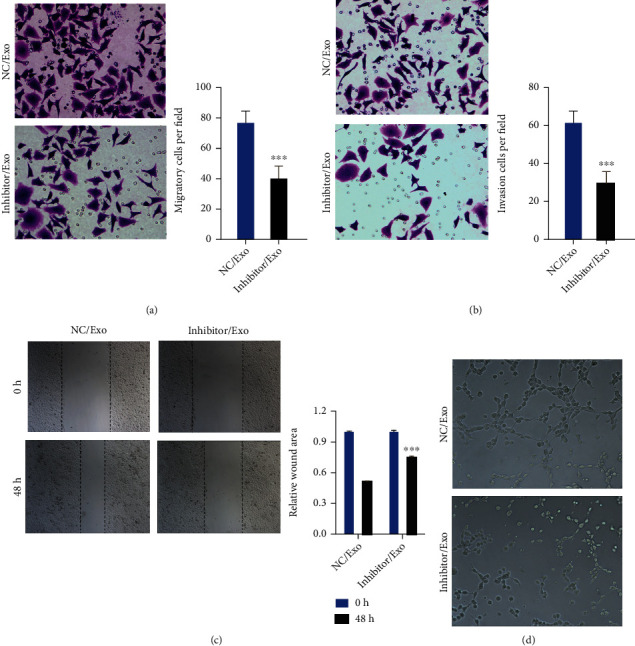
miR-4454 inhibitor-mediated exosomes significantly prevented migration, invasion, and vascularization of HepG2 cells. HepG2 cells were processed with exosomes from HepG2 cells after miR-4454 inhibitor transfection. (a, b) The influences of miR-4454 inhibitor-mediated exosomes on the migration and invasion of HepG2 cells were assessed by Transwell assay. (c) Cellular migration ability was assayed by a wound healing assay. (d) Cellular angiogenic ability was certified on Matrigel. ^∗∗∗^*p* < 0.001.

And Transwell results showed that miR-4454 inhibitor-mediated exosomes had strong inhibitory effects on HepG2 cell migration and invasion (*p* < 0.001, Figures 10(a) and 10(b)). The wound healing assay data also verified the inhibiting effect of miR-4454 inhibitor-mediated exosomes on HepG2 cell migration (*p* < 0.001, Figure 10(c)). Simultaneously, miR-4454 inhibitor-mediated exosomes could inhibit the vascularization of HepG2 cells (Figure 10(d)). Therefore, we showed that miR-4454 inhibitor-mediated exosomes, such as the miR-4454 inhibitor, could also memorably suppress the progress of HCC.

## 4. Discussion

Cell canceration is caused by protooncogene and antioncogene mutations in cells under the action of carcinogenic factors [29]. External factors in cell carcinogenesis include physical, chemical, and viral carcinogens [30]. Plentiful studies have testified that external stimuli, such as high temperature, hypoxia, low/high glucose, changes in calcium concentration, nutrient deficiency, acidosis, and heavy metals, can improve cell proliferation capacity [31, 32]. In addition, continuous stress conditions can lead to the initiation of the apoptotic process [33, 34]. However, the effects of external stimuli on cancer cell-derived exosomes are not yet completely clear. In our study, the exosomes were extracted from HepG2 cells under heat shock, TGF-*β*1, doxorubicin, acidic, and H/R conditions, and we discovered that the morphology of the exosomes from HepG2 cells was observably different under different external stimuli, suggesting that external stimuli could affect the morphology of exosomes from HepG2 cells.

Research has revealed that miRNA expression profiles in exosomes were significantly different from those of derived cells, suggesting that miRNAs could selectively enter exosomes [35–37]. Our previous study also revealed that HCC-derived exosomes contain cancer-inhibiting miRNAs with a much higher abundance than HCC tissues. However, it is unclear whether HCC cells could exhale some cancer-inhibiting miRNAs that limit their own development by exosomes. Furthermore, due to the plasticity of the tumor, it constantly adjusts to adapt to changes in the environment during its occurrence and development, which are closely related to changes in the miRNA expression profile [38, 39]. Our study first adopted RNA sequencing to analyze miRNAs in exosomes of HepG2 cells under heat shock, TGF-*β*1, doxorubicin, acidic, and H/R conditions. And the results provided a large number of differentially expressed miRNAs in exosomes, which were associated with heat shock, TGF-*β*1, doxorubicin, acidic, and H/R. Meanwhile, we provided abundant miRNA enrichment pathways in the BP, CC, and MF terms. Furthermore, through verification experiments, we discovered multiple differentially expressed miRNAs, including miR-96a-5p, miR-381-3p, miR-582-5p, miR-4454, miR-7975 and miR-16-5p, miR-4532, miR-4800-3p, miR-1307-5p, and miR-3168 in exosomes from HepG2 cells after stimulations, especially miR-4454.

Molecules that can regulate exosome secretion have been reported to include endosomal sorting complexes required for transport complexes (ESCRT), vacuolarproteinsorting4 (Vps4) complexes, Alix, and Rab enzyme families [40]. The ESCRT complex is composed of ESCRT-0 (Hrs and STMA1/2 subunits), ESCRT-I (Tsg101, Vps28, and Vps37A/B/C/D subunits), ESCRT-II (Vps22, Vps25, and Vps36 subunits), and ESCRT-III (Vps2A/B, Vps24, CHMP1A/B, and chmp5/6/7 subunits) [41]. Exosome biosynthesis is closely related to the ESCRT complex, and the absence of components (Hrs) can lead to a decrease in exosome secretion [42]. Vps4, as one of the key regulators of the activity of the ESCRT complex, can regulate exosome size and the formation rate of exosomes [43]. As a bridging factor connecting ESCRT-I and ESCRT-III, Alix can be used as a marker for exosomes [44]. Rab27a and Rab27b in the Rab enzyme family are also associated with exosome secretion regulation [45]. In our study, we discovered that doxorubicin could upregulate. At the same time, TGF-*β*1 could downregulate Vps4A, Rab27A, Alix, and Hrs expressions in HepG2 cells and exosomes, suggesting that doxorubicin could promote exosome secretion, while TGF-*β*1 could inhibit exosome secretion in HepG2 cells. Furthermore, we certified that Vps4A and Rab27A were the target genes of miR-4454 and could be downregulated by miR-4454 in HepG2 cells. Furthermore, we demonstrated that the miR-4454 inhibitor could significantly reduce miR-4454 expression in HepG2 cells, while the miR-4454 inhibitor could significantly increase miR-4454 expression in exosomes, indicating that the miR-4454 inhibitor could promote miR-4454 secretion in exosomes. Furthermore, functional experiments revealed that both miR-4454 inhibitor and miR-4454 inhibitor-mediated exosomes could markedly suppress proliferation, migration, invasion, and vascularization and prominently accelerate cycle arrest, apoptosis, and ROS of HepG2 cells.

## 5. Conclusions

**Figure 11:**
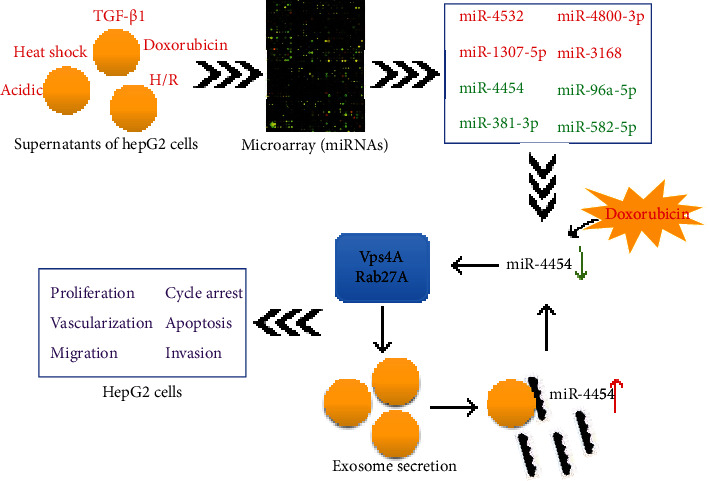
Through the selection and identification of differentially expressed miRNAs in exosomes after stimulation (heat shock, TGF-*β*1, doxorubicin, acidic, and H/R), we discovered that doxorubicin could cause a decrease in miR-4454 to promote Vps4A and Rab27A expressions, which could then induce exosome secretion and enhance the miR-4454 content in exosomes, thus accelerating the progression of liver carcinoma.

In summary, for the first time, we identified a mass of upregulated and downregulated miRNAs in exosomes of HepG2 cells under heat shock, TGF-*β*1, doxorubicin, acidic, and H/R conditions by RNA sequencing. Our study was also the first to verify that external stimuli, such as doxorubicin and TGF-*β*1, can affect exosome secretion in HepG2 cells. Furthermore, we testified that inhibition of screened miR-4454 and miR-4454 inhibitor-mediated exosomes could inhibit HCC progression by targeting Vps4A and Rab27A (Figure 11). Therefore, miR-4454 and miR-4454-mediated exosomes could play an essential role in HCC.

## Figures and Tables

**Table 1:** The primer sequences for qRT-PCR assay.

Name	Sequence (5′-3′)
U6 F	CTCGCTTCGGCAGCACA
U6 R	AACGCTTCACGAATTTGCGT
All R	CTCAACTGGTGTCGTGGA
miR-96a-5p RT	CTCAACTGGTGTCGTGGAGTCGGCAATTCAGTTGAGAGCAAAAAT
miR-96a-5p F	ACACTCCAGCTGGGTTTGGCACTAGCACATT
miR-381-3p RT	CTCAACTGGTGTCGTGGAGTCGGCAATTCAGTTGAGACAGAGAGC
miR-381-3p F	ACACTCCAGCTGGGTATACAAGGGCAAGCT
miR-582-5p RT	CTCAACTGGTGTCGTGGAGTCGGCAATTCAGTTGAGAGTAACTGG
miR-582-5p F	ACACTCCAGCTGGGTTACAGTTGTTCAACCA
miR-4454 RT	CTCAACTGGTGTCGTGGAGTCGGCAATTCAGTTGAGTGGTGCCG
miR-4454 F	ACACTCCAGCTGGGGGATCCGAGTCACGGC
miR-1273e RT	CTCAACTGGTGTCGTGGAGTCGGCAATTCAGTTGAGTCCACTT
miR-1273e F	ACACTCCAGCTGGGTTGCTTGAACCCAGGAAG
miR-663a RT	CTCAACTGGTGTCGTGGAGTCGGCAATTCAGTTGAGGCGGTCC
miR-663a F	ACACTCCAGCTGGGAGGCGGGGCGCCGCGGGA
miR-1307-5p RT	CTCAACTGGTGTCGTGGAGTCGGCAATTCAGTTGAGAGCCGGT
miR-1307-5p F	ACACTCCAGCTGGGTCGACCGGACCTCGACC
miR-4532 RT	CTCAACTGGTGTCGTGGAGTCGGCAATTCAGTTGAGCGCCGGG
miR-4532 F	ACACTCCAGCTGGGCCCCGGGGAGCCC
miR-4800-3p RT	CTCAACTGGTGTCGTGGAGTCGGCAATTCAGTTGAGGTGGACA
miR-4800-3p F	ACACTCCAGCTGGGCATCCGTCCGTCTGT
miR-3168 RT	CTCAACTGGTGTCGTGGAGTCGGCAATTCAGTTGAGGTCTGAC
miR-3168 F	ACACTCCAGCTGGGGAGTTCTACAGTC
miR-16-5p RT	CTCAACTGGTGTCGTGGAGTCGGCAATTCAGTTGAGCGCCAAT
miR-16-5p F	ACACTCCAGCTGGGTAGCAGCACGTAAATATT
miR-7975 RT	CTCAACTGGTGTCGTGGAGTCGGCAATTCAGTTGAGTGGTGCCGT
miR-7975 F	ACACTCCAGCTGGGATCCTAGTCACG
GAPDH F	TGTTCGTCATGGGTGTGAAC
GAPDH R	ATGGCATGGACTGTGGTCAT
VPS4A F	CCACGCTATCAAGTATGAGGC
VPS4A R	CCGTGTTTCTCTTTGCTTCGTA
RAB27A F	GCTTTGGGAGACTCTGGTGTA
RAB27A R	TCAATGCCCACTGTTGTGATAAA
BAG5 F	AGTTATCGGCTTCAGTGGTCT
BAG5 R	CTGCCCGCTTCCTAGCTTG
TRPV6 F	ACTGACCTCGACTCTCTATGAC
TRPV6 R	GTGGTGATGATAAGTTCCAGCAG

## Data Availability

The data used to support the findings of this study are included within the article.
